# Significance of CAVI as a Functional Stiffness Parameter: Beyond the Prognostic Value for Cardiovascular Events

**DOI:** 10.1016/j.jacadv.2024.101018

**Published:** 2024-06-03

**Authors:** Daiji Nagayama, Kohji Shirai, Atsuhito Saiki

**Affiliations:** a Department of Internal Medicine, Nagayama Clinic, Tochigi, Japan; b Center of Diabetes, Endocrinology and Metabolism, Sakura Medical Center, Toho University, Chiba, Japan; c Department of Internal Medicine, Mihama Hospital, Chiba, Japan

**Keywords:** arterial stiffness, cardio-ankle vascular index, prognostic value, ventricular-arterial interaction

**Figure:**
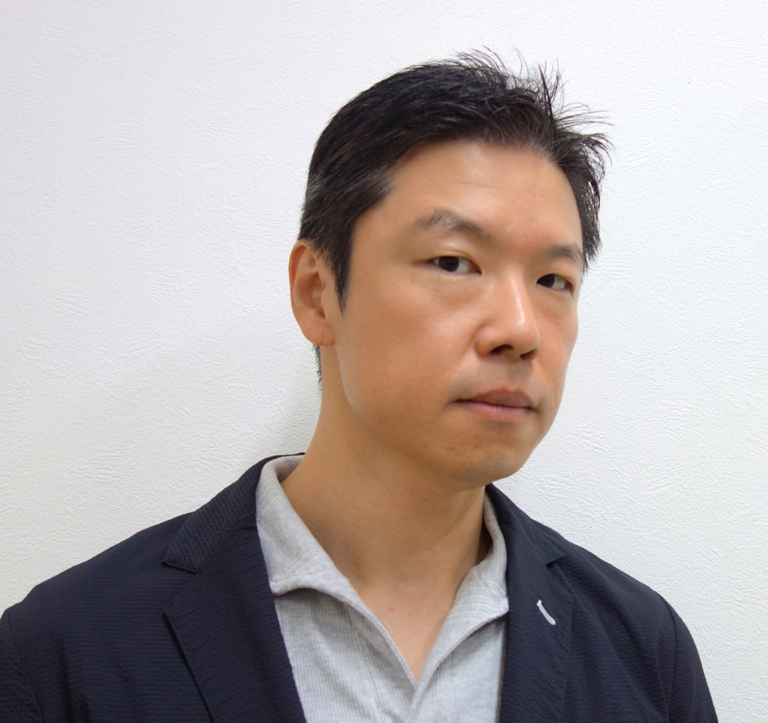

Cardio-ankle vascular index (CAVI) is an arterial stiffness parameter that includes the entire arterial tree from the aortic valve to the tibial artery and is derived from stiffness parameter β.^1^ CAVI has been theoretically and clinically proven to be independent of blood pressure (BP) at the time of measurement, a characteristic not found in conventional parameters. For example, unlike alpha1-adrenergic receptor blocker, metoprolol as a selective beta1-adrenergic receptor blocker does not reduce CAVI immediately after administration.^2^ This suggests that CAVI is not affected by immediate changes in BP, while decreasing with vascular smooth muscle relaxation. In this issue of *JACC: Advances*, Tavolinejad et al^3^ report on a meta-analysis examining the prognostic value of CAVI. The systematic review included 32 studies (N = 105,845) and demonstrated that the baseline CAVI was independently associated with future cardiovascular (CV) and kidney outcomes. Notably, the prognostic value of CAVI was confirmed in a multinational setting and was present even when limited to the primary prevention group.

The role of CAVI as a structural stiffness parameter is well established, and many prospective studies have proposed a CAVI of 9.0 or higher as the optimal cutoff to predict CV disease (CVD).^4^ CAVI has recently been shown to have significance beyond its prognostic value for CV events (Figure 1). The present editorial highlights new horizons for vascular function research utilizing CAVI.

**Figure 1:**
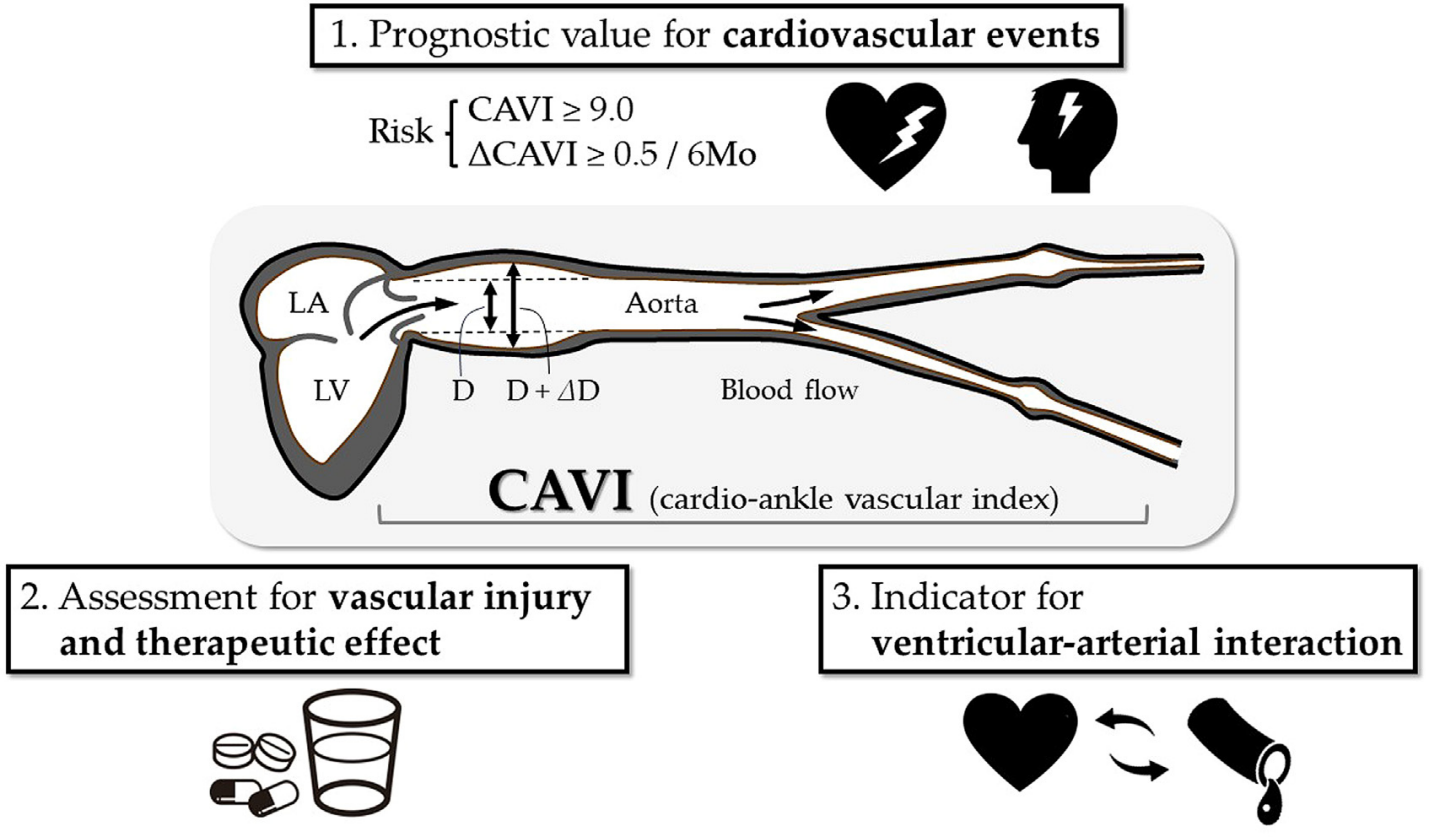
**Significance of CAVI Beyond Prognostic Value of Cardiovascular Events** CAVI = cardio-ankle vascular index; LA = left atrium; LV = left ventricle.

The proposed pathophysiology is that vasa vasorum and medial smooth muscle contraction, indicated by a rapid rise in CAVI, are involved in plaque rupture.^5^ Shimizu et^5^ al reported that healthy individuals showed a transient increase in CAVI immediately after the Great East Japan Earthquake in 2011. An increase in CV events and mortality was observed during the same period, suggesting that psychological stress might have triggered CV events via rapid deterioration of vascular function. Furthermore, cases of myocardial infarction, cerebral hemorrhage, or aortic dissection following a short-term upward trend in CAVI have also been reported, respectively. It is speculated that a rapid rise in CAVI means medial smooth muscle contraction and strangling vasa vasorum, lead to ischemia of vulnerable plaque. An increase in CAVI of more than 0.5 over a 6-month may be a warning sign. CAVI is considered to reflect not only structural stiffness but also functional stiffness and should be continuously monitored in individuals with CVD risks.

The value of CAVI in daily clinical practice is not limited to predicting CV events. CAVI reflects the severity of CV risks including glucose intolerance, hypertension, dyslipidemia, sleep apnea, and smoking.^4^ Additionally, appropriate therapeutic interventions can reduce CAVI, suggesting that CAVI can help assess vascular injury and therapeutic effects. Metabolic syndrome (MetS) is also associated with increased CAVI, while, contrary to intuition, body mass index and waist circumference (WC) are known to have an inverse relationship with CAVI.^6^ This obesity paradox may be because body mass index and WC preferentially reflect vascular protective body composition such as subcutaneous fat and/or skeletal muscle mass. We have reported that “a body shape index (ABSI)” is the abdominal obesity index most strongly associated with vascular injury, that is, increased CAVI, attributable to abdominal obesity. In addition, ABSI reflects not only visceral fat accumulation but also sarcopenia and is associated with CAVI independently of conventional metabolic parameters. It has also been shown that adopting high ABSI instead of high WC for MetS diagnosis reinforces the identification of individuals at risk for high CAVI and kidney function decline. This modification of the ABSI-based MetS diagnostic criteria will eventually need to be validated also in terms of CVD risk assessment.

Finally, we emphasize the significance of CAVI as an assessment tool for ventricular-arterial interaction. The independency of CAVI from BP at the measuring time allows real-time assessment of vascular alterations in response to hemodynamic change. Several studies shown that elevated CAVI is independently associated with left ventricular diastolic dysfunction in individuals with preserved systolic function.^7^ Zhang et al^8^ reported that the decrease in CAVI was associated with the increase in left ventricular ejection fraction in individuals recovering from acute heart failure. These findings suggest that increased left ventricular afterload, as indicated by increased CAVI, is a reversible regulator for cardiac dysfunction. In addition, ventricular-arterial interaction may also be mediated by pulmonary arterial hypertension. Sato et al^9^ reported that balloon pulmonary angioplasty for chronic thromboembolic pulmonary hypertension reduces CAVI. The change in CAVI after the angioplasty was associated not only with the change in mean pulmonary artery pressure but also with changes in biventricular volumes, that is, cardiac remodeling. This suggests that chronic thromboembolic pulmonary hypertension-induced pathophysiology including hypoxia, left ventricular compression, and pulmonary hypertension may affect the ventricular-arterial interaction. Furthermore, the increase in CAVI associated with decreased circulating plasma volume is further evidence to supporting interactions. In hemodialysis patients, blood removal causes a transient increase in CAVI accompanied by a decreased BP, which returns to baseline by blood return.^10^ This reactive alteration in arterial stiffness may be aimed at maintaining blood flow to major organs via central volume shift. Similar finding has also been observed in an experimental rabbit model, and Nagasawa et al^11^ showed that this phenomenon may be induced via non-neuronal cellular factors, for example, mechanogated ion channels in the arterial smooth muscle, rather than the autonomic nervous system. In contrast, there are pathophysiologies in which CAVI is reduced even with decreased circulating plasma volume. We previously reported that CAVI was extremely low in the acute phase of sepsis, especially in severe cases, and raised after treatment.^12^ Since sepsis patients did not show significant BP change before and after treatment, CAVI might reflect sepsis-induced vascular alteration which is not indicated by BP change. In other words, the decreased CAVI without hypotension in the acute phase of sepsis might indicate a compensatory increase in cardiac output to maintain BP.

The arterial stiffness parameters were initially developed to simply assess structural stiffness. However, through attempt to correct for the BP dependence, the ability to assess functional stiffness has also been acquired. As described in this editorial, recent studies on CAVI have shown that the vessels per se actively and autonomously regulate their elasticity, providing evidence for ventricular-arterial interaction. These findings may open new horizons in the vascular function research.

## Funding support and author disclosures

The authors have reported that they have no relationships relevant to the contents of this paper to disclose.
